# Loss of *Caspase-3* sensitizes colon cancer cells to genotoxic stress via RIP1-dependent necrosis

**DOI:** 10.1038/cddis.2015.104

**Published:** 2015-04-23

**Authors:** M F Brown, B J Leibowitz, D Chen, K He, F Zou, R W Sobol, D Beer-Stolz, L Zhang, J Yu

**Affiliations:** 1 Department of Pathology, University of Pittsburgh School of Medicine, 5117 Centre Ave., Pittsburgh, PA, USA; 2 University of Pittsburgh Cancer Institute, Hillman Cancer Center Research Pavilion, 5117 Centre Ave., Pittsburgh, PA, USA; 3 Department of Pharmacology & Chemical Biology, University of Pittsburgh School of Medicine, 5117 Centre Ave., Pittsburgh, PA, USA

## Abstract

Caspase-3 is the best known executioner caspase in apoptosis. We generated caspase-3 knockout (C3KO) and knockdown human colorectal cancer cells, and found that they are unexpectedly sensitized to DNA-damaging agents including 5-fluorouracil (5-FU), etoposide, and camptothecin. C3KO xenograft tumors also displayed enhanced therapeutic response and cell death to 5-FU. C3KO cells showed intact apoptosis and activation of caspase-7 and -9, impaired processing of caspase-8, and induction of necrosis in response to DNA-damaging agents. This form of necrosis is associated with HMGB1 release and ROS production, and suppressed by genetic or pharmacological inhibition of RIP1, MLKL1, or caspase-8, but not inhibitors of pan-caspases or RIP3. 5-FU treatment led to the formation of a z-VAD-resistant pro-caspase-8/RIP1/FADD complex, which was strongly stabilized by caspase-3 KO. These data demonstrate a key role of caspase-3 in caspase-8 processing and suppression of DNA damage-induced necrosis, and provide a potentially novel way to chemosensitize cancer cells.

Colorectal cancer is a major cancer killer in the United States and worldwide.^1^ Chemotherapeutic agents such as 5-fluorouracil (5-FU) and irinotecan (Camptosar) are commonly used in treating patients with colon cancer and other solid tumors. However, the 5-year survival of colon cancer patients with advanced diseases is <10% even with aggressive treatments.^1^ Most conventional chemotherapeutic agents cause DNA damage and trigger apoptosis,^2^ which is regulated by mitochondria-dependent intrinsic and death receptor-dependent extrinsic apoptotic pathways converging on the activation of executioner caspases-3 and -7.^2^ During transformation, neoplastic cells frequently become resistant to apoptosis via genetic and epigenetic mechanisms, driving accumulation of additional oncogenic events, and therapeutic resistance.^3^ Therefore, the exploration of alternative death pathways might provide new therapeutic options.

Necrosis has long been viewed as an unregulated form of cell demise that promotes inflammation and tissue damage, whereas emerging evidence indicates that some forms of necrosis are programmed.^4, 5^ They can be initiated upon activation of the extended TNF-*α* receptor family at the cell surface, propagated through the receptor-interacting serine–threonine kinases, RIP1 and RIP3, and actively suppressed by apoptosis.^6, 7, 8, 9^ In mice, loss of caspase-8 leads to RIP3-dependent necrosis and embryonic lethality,^10, 11^ or intestinal inflammation involving TNF-*α* production.^12, 13^ In HT29 colon cancer cells, the addition of pan-caspase inhibitor z-VAD switches TNF-*α* and SMAC mimetic-induced apoptosis to RIP1/RIP3-dependent necrosis via downstream effector proteins mixed lineage kinase domain-like protein (MLKL) and phosphoglycerate mutase family member 5 (PGAM5).^14, 15^ Induction of programmed necrosis, or necroptosis, is stimuli- and cell type-dependent, and can also occur independent of either RIP1, RIP3,^6, 16, 17^ or both.^18^ The role and regulation of necrosis following DNA damage in relation to therapeutic outcomes has remained largely unexplored.^8, 9^

In the current study, we report an unexpected function of caspase-3 in suppressing necrosis triggered by DNA-damaging agents in colon cancer cells. Caspase-3 knockout (C3KO) or knockdown (KD) colon cancer cells showed normal apoptotic response, but increased sensitivities to DNA-damaging agents in cell culture and in mice, at least in part, via RIP1-, and caspase-8-dependent necrosis. Our findings provide a potentially novel approach to chemosensitize cancer cells.

## Results

### C3KO cells are more sensitive to DNA-damaging agents in culture and in mice

**Figure 1:**
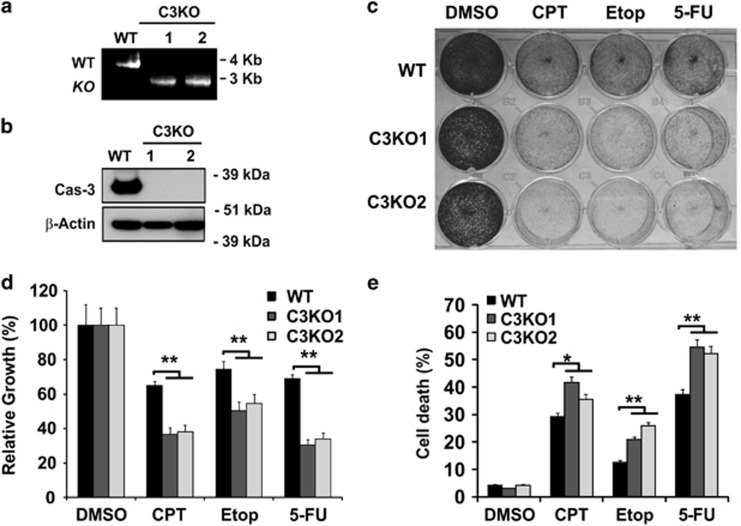
*Caspase-3* knockout cells are more sensitive to DNA-damaging agents. (a) PCR analysis showing both full-length (WT) and targeted *caspase-3* allele (C3KO). (**b**) Protein expression of caspase-3 in HCT116 WT and two independent C3KO clones. Molecular weight markers are indicated on the right. (**c**) Adherent cells in HCT116 WT and two independent C3KO clones stained by crystal violet 48 h after treatment with camptothecin (500 nM), etoposide (50 *μ*M), or 5-FU (50 *μ*g/ml). (**d**) Cell proliferation measured by MTS assay in cells as treated in (**c**). (**e**) Cell death in cells as treated in (**c**), including fractions of either PI+ or Annexin V+, or both (PI+/Annexin V+) populations. (**d, e**) Data are the mean + S.E.M. of triplicate wells. **P<0.01

The human colon cancer cell line HCT116 has been extensively used to study apoptotic responses to anticancer agents, and is amenable for gene targeting.^19^ In an attempt to model apoptosis resistance, we generated several C3KO HCT116 cell lines using the recombinant adeno-associated virus (rAAV) system (Supplementary Figure S1A). Deletion of exon 5–7 of *Caspase-3* gene and loss of protein were verified by polymerase chain reaction (PCR) and western blot (Figures 1a and b), respectively. Unexpectedly, independent C3KO lines were found to be significantly more sensitive to different classes of DNA-damaging agents including camptothecin (CPT), etoposide (Etop) and 5-flurouracil (5-FU) (Figure 1c), evident by reduced cell proliferation (Figure 1d) and increased cell death (Figure 1e).

**Figure 2:**
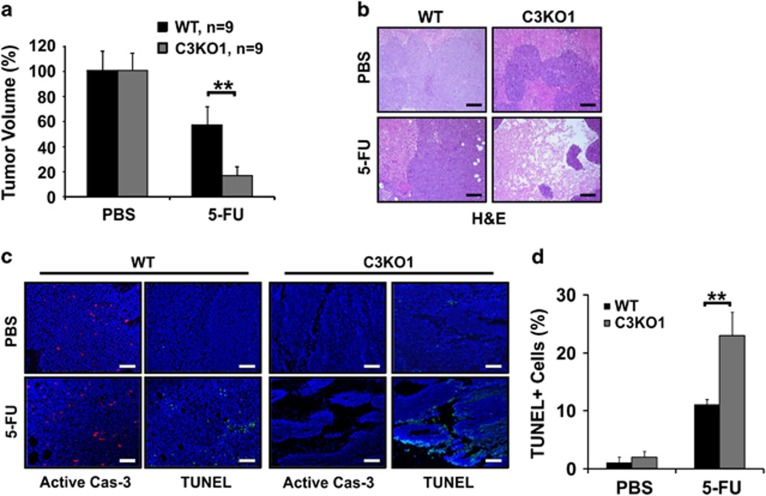
*Caspase-3* KO tumors show increased response to 5-FU *in vivo*. (**a**) The relative growth of WT and C3KO tumors in PBS and 5-FU groups. 5-FU (50 mg/kg) was given intraperitoneally (IP) every other day for 14 days when tumors reached ~50 mm^3^ (day 0). *N*=9/group. (**b**) H&E staining of tumors with indicated genotypes after the last treatment. (**c**) Staining of active caspase-3 and TUNEL in the tumors after the last treatment. Scale bars, 200 *μ*m. (**d**) Quantification TUNEL-positive cells in HCT116 WT and C3KO tumors in (**c**). Data are the mean + S.E.M. of three randomly chosen tumors in each group. **P<0.01

In xenograft model, C3KO tumors responded significantly better than WT tumors to 5-FU, showing a greater growth suppression (Figure 2a). C3KO tumors exhibited extensive loss of cellularity in multiple regions and elevated TUNEL positivity while remained negative for active caspase-3 (Figures 2b and d; Supplementary Figures S1B and S1C). These results indicate that loss of *caspase-3* enhances tumor response and cell killing to DNA-damage agents *in vitro* and *in vivo*.

### C*aspase-*3 KO cells show enhanced cell death with intact apoptosis after DNA damage

**Figure 3:**
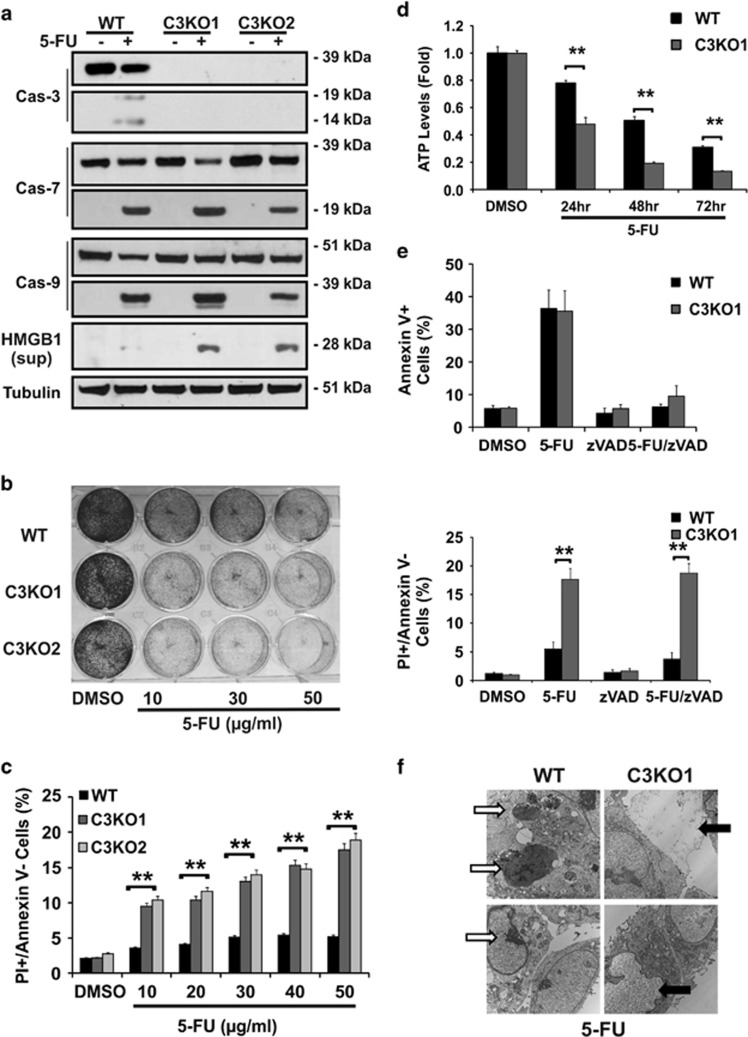
Induction of necrosis in *caspase-3* KO cells by DNA-damaging agents. HCT116 WT and C3KO cells were treated with DMSO or 5-FU (50 *μ*g/ml or as specified). (**a**) Levels of caspases, RIP1, and HMGB1 in the culture medium in WT and three independent C3KO lines 24 h. (**b**) Adherent cells stained by crystal violet 48 h after the indicated doses. (**c**) Fractions of PI+/Annexin V-(non-apoptotic dying) cells at 48 h at the indicated doses. (**d**) Cellular ATP levels at the indicated times. Values were normalized to the DMSO-treated cells. (**e**) Fractions of Annexin V+ or PI+/Annexin V- cells 48 h with or without z-VAD (20 *μ*M). (**f**) Representative images of transmission electron microscopy of cells at 24 h. White arrows denote apoptotic cells, black arrows denote necrotic cells. (**c**–**e**) Data are the mean + S.E.M. of triplicate wells. **P<0.01

We further analyzed cell death in C3KO and WT HCT116 cells after 5-FU treatment. C3KO cells showed intact activation of caspase-7 and -9 (Figure 3a), induction of DNA damage response markers such as *γ*-H2AX, phosphorylated p53 (S15), p53 targets, and cIAP1 depletion (Supplementary Figure S2A). In addition, C3KO cells showed signs of non-apoptotic cell death, including extracellular release of nuclear protein HMGB1^20^ (Figure 3a), and dose-dependent increase in cell loss and PI+/Annexin V- populations, and time-dependent reduction in cellular ATP levels (Figures 3b–d).

5-FU treatment induced comparable and high levels of apoptosis, or Annexin V+ populations, in both C3KO and parental cells, which were blocked by the pan caspase-inhibitor z-VAD (Figure 3e upper, Supplementary Figure S2B). In contrast, z-VAD-resistant PI+/Annexin V- (non-apoptotic), or PI+ populations were significantly elevated in C3KO cells compared with parental cells (Figure 3e; Supplementary Figure S2C). Transmission electron microscopy (EM) readily detected cells with classical necrotic morphology such as disintegration of nuclear and plasma membrane and leakage of nuclear and cellular content in 5-FU-treated C3KO cells, which were rare in parental cells (Figure 3f, black arrows). As expected, cells showing classical apoptotic features such as condensed and fragmented nuclei were more prominent in 5-FU-treated C3KO and parental cells (Figure 3f, white arrows). HMGB1 release and PI+/Annexin V- populations were also strongly induced in CPT or Etop-treated independent C3KO clones compared with WT cells (Supplementary Figures S2D and S2E). These findings demonstrate that caspase-3 is dispensable for apoptosis induced by DNA-damaging agents, and its loss activates non-apoptotic cell death.

### Loss of caspase-3 stabilizes a z-VAD-resistant pro-caspase-8/RIP1/FADD complex

**Figure 4:**
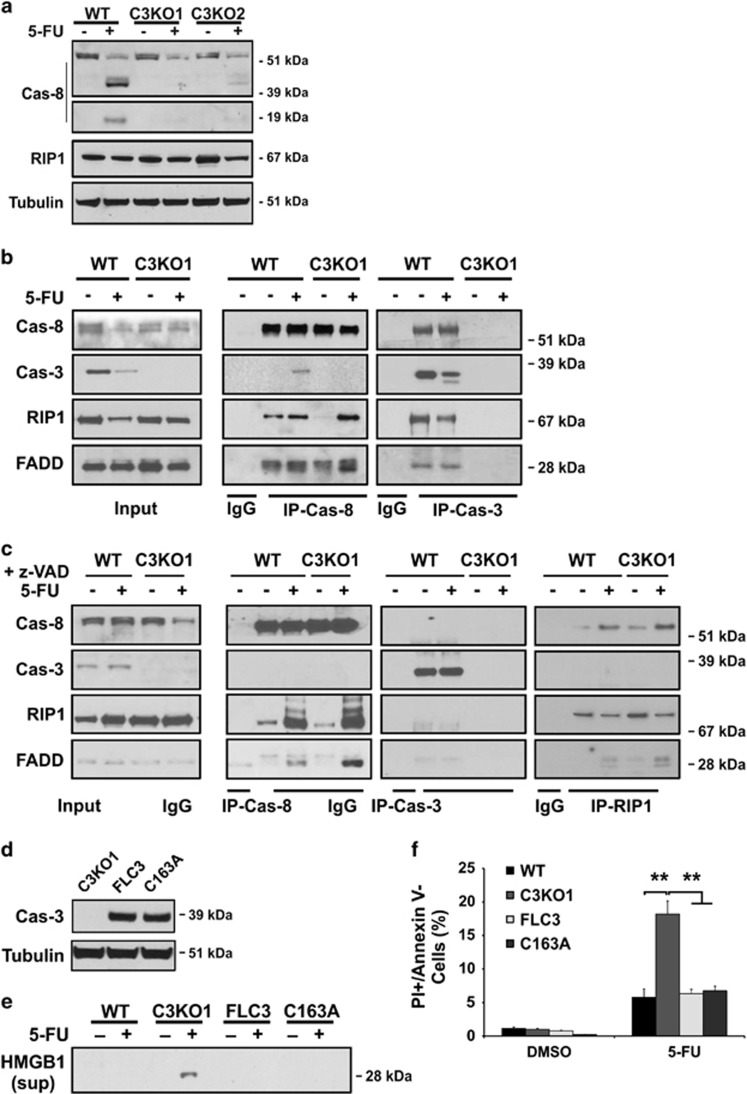
Formation of a z-VAD-resistant pro-casapse-8 complex after 5-FU treatment. HCT116 or C3KO cells were treated with DMSO or 5-FU (50 *μ*g/ml). (**a**) Levels of caspase-8 and RIP1 at 24 h. (**b**) Analysis of caspase-8 (IP-Cas-8) or caspase-3 interacting proteins (IP-Cas-3) at 24 h. Input, whole cell lysate. (**c**) Analysis of caspase-8 (IP-Cas-8), caspase-3 (IP-Cas-3), or RIP1 (IP-RIP1) interacting proteins. Cells treated as (**b**) in the presence of z-VAD (20 *μ*M). (**d**) Reconstitution of catalytically dead (C163A) or full-length caspase-3 (FLC3) in C3KO cells confirmed by western blotting. (**e**) Levels of HMGB1 in the medium of reconstituted C3KO cells at 48 h. (**f**) Fractions of PI+/Annexin V- in the reconstituted C3KO cells at 48 h. Data are the mean + S.E.M. of triplicate wells. **P<0.01

The ripoptosome, a caspase-8 and RIP1-containing complex, has been suggested to promote necrosis in some cells if caspase-8 activity is inhibited.^11, 21^ The processing of caspase-8 was profoundly reduced in C3KO cells compared with parental cells (Figure 4a). We first used immunoprecipitation (IP) to examine potential interaction between caspase-8 and RIP1 by caspase-8 pull-down (IP-Cas-8). We found that 5-FU treatment promotes the assembly of a pro-caspase-8/RIP1/FADD complex, and caspase-3 recruitment to this complex in WT cells. The complex formation was enhanced in the C3KO cells (Figure 4b). Reciprocal caspase-3 pull-down (IP-Cas-3) confirmed the highly specific binding of the full-length and partially processed caspase-3 to this complex in only WT cells (Figure 4b). In untreated WT cells, the lack of caspase-3 detection in caspase-8 pull-down (IP-Cas-8) might be explained by a weak or transient binding, which is likely stabilized on the addition of caspase-3 antibody (Figure 4b, IP-Cas-8 *versus* IP-Cas-3), and/or only a minor fraction of RIP1 complexed with caspase-3.

C3KO cells showed substantially higher levels of z-VAD-resistant cell death after 5-FU treatment, we therefore determined the effects of z-VAD on complex formation. The z-VAD treatment blocked RIP1 decrease or caspase-3 cleavage, strongly enhanced the complex formation in WT and C3KO cells (IP-Cas-8), but unexpectedly blocked caspase-3 recruitment completely (Figure 4c, IP-Cas-8). Reciprocal IP-Cas-3 or RIP1 confirmed the loss of caspase-3 binding to the complex, but enhanced RIP1 binding with caspase-8/FADD in C3KO cells (Figure 4c). Quantification of 5-FU-induced interactions indicated a significant increase of RIP1 binding to caspase-8 (from 1.23 to 10.47) in C3KO cells, and a further increase in FADD to RIP1 ratio (from 1.82 : 10.47 to 7.5 : 7.3) with z-VAD (Supplementary Table S2 and Supplementary Table S3). Interestingly, z-VAD treatment caused a prominent laddering of RIP1 only in 5-FU-treated cells (Figure 4c, IP-Cas-8), indicative of post-translational modification. Using reconstitution experiments, we found that stable expression of the catalytically dead caspase-3 mutant (C163A) in C3KO cells rescued HMGB1 release and PI+/Annexin V- populations, as well as the full-length caspase-3 (Figures 4d and f). These data strongly suggest that caspase-3 facilitates caspase-8 and RIP1 cleavage upon DNA damage, and in turn destroys the pro-caspase-8/RIP/FADD complex. The scaffold function of caspase-3 might allow other caspases to cleave or activate caspase-8 or RIP1.

### 5-FU promotes RIP1- and Caspase-8-dependent necrosis in *caspase-3* KO cells

**Figure 5:**
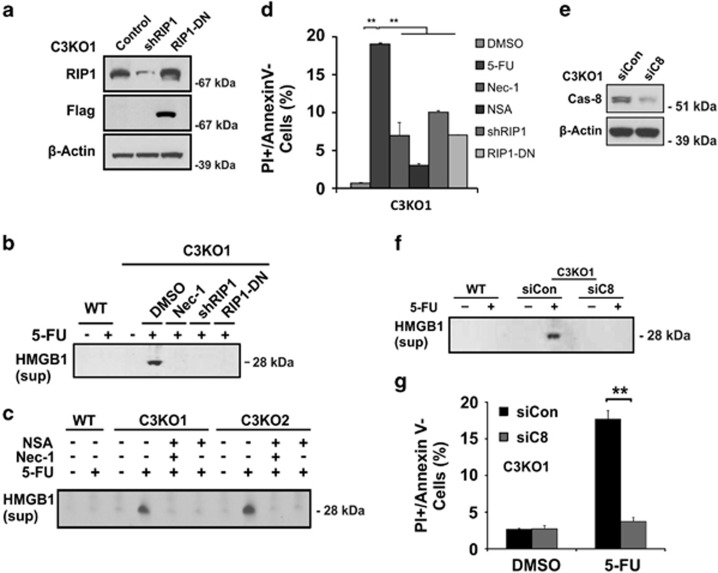
5-FU induces RIP1-dependent necrosis in *caspase-3* KO cells. C3KO or derived cells were treated with 5-FU (50 *μ*g/ml). (**a**) Stable expression of shRIP1 or dominant-negative RIP1 (Flag-RIP1-DN) in C3KO1 cells was confirmed by western blotting. (**b**) Levels of HMGB1 release (medium) at 48 h with or without RIP1 inhibition. Necrostatin-1 (Nec-1, 15 *μ*M). (**c**) Levels of HMGB1 release (medium) at 48 h with inhibitors of RIP1 (Nec-1) or MLKL (necrosulfonamide, NSA, 2 *μ*M) in two C3KO lines. (**d**) Fractions of PI+/Annexin- C3KO1 cells at 48 h without or with Nec-1, NSA, ShRIP1, RIP1-DN. (**e**) Knockdown of *caspase-8* in C3KO1 cells by siRNA confirmed by western blotting. (**f**) Levels of HMGB1 (medium) from C3KO1 cells with or without *caspase-8* siRNA 48 h after 5-FU treatment. (**g**) Fractions of PI+/Annexin V-cells as treated in (**c**). (**d** and **g**) Data are the mean + S.E.M. of triplicate wells. **P<0.01

We then investigated whether RIP1 or caspase-8 are required for 5-FU-induced necrosis in C3KO cells using z-VAD insensitive HMGB1 release and PI+/Annexin- populations as major markers. 5-FU-induced HMGB1 release (Figures 5b–c) and the PI+/Annexin V- population (Figure 5d) in C3KO cells were significantly suppressed when RIP1 was inhibited by short-hairpin RNA (shRNA), kinase domain deleted RIP1, or the small molecule inhibitor necrostatin-1 (Nec-1).^22^ The MLKL inhibitor necrosulfonamide (NSA)^15^ also strongly suppressed these markers in two C3KO clones (Figures 5c and d). None of these agents blocked 5-FU-induced activation of caspase-7 (Supplementary Figure S3). *Caspase-8* KD by siRNA also significantly reduced 5-FU-induced HMGB1 release and PI+/Annexin V- populations in C3KO cells (Figures 5e and g). These findings demonstrated that RIP1 and pro-caspase-8 are required for 5-FU-induced necrosis in C3KO cells.

### *Caspase-3* deficiency promotes DNA-damage-induced and RIP3-independent necrosis in colon cancer cells

**Figure 6:**
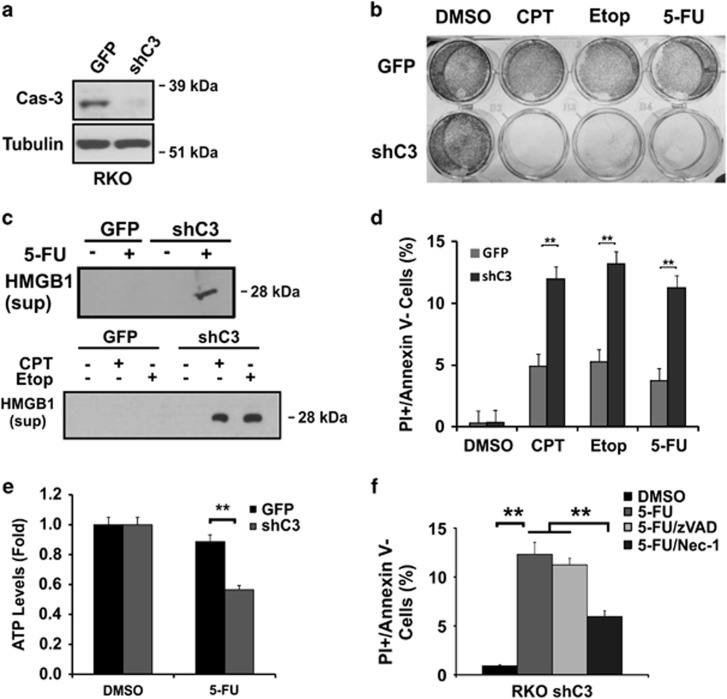
RKO caspase-3 KD cells are sensitized to DNA-damaging agents. (**a**) Stable *caspase-3* knockdown (shC3) RKO cells confirmed by western blotting. (**b**) Crystal violet staining of adherent RKO GFP or shC3 cells 48 h after CPT (500 nM), Etoposide (50 *μ*M) or 5-FU (50 *μ*g/ml) treatment. (**c**) Levels of HMGB1 in the medium from cells at 48 h with or without 5-FU treatment. (**d**) Fractions of PI+/Annexin V- cells as treated in (**c**). (**e**) ATP levels in cells as treated in (**c**). (**f**) Fractions of PI+/Annexin V- cells 48 h after 5-FU treatment, with or without z-VAD (20 *μ*M) or Nec-1 (15 *μ*M). (**d**–**f**) The data are mean + S.E.M. of triplicate wells. **P<0.01

To rule out cell line-specific induction of necrosis upon *caspase-3* ablation, we generated stable RKO and HCT116 *caspase-3* KD lines using shRNA (shC3) (Figure 6a; Supplementary Figure S4A). RKO C3KD cells showed increased sensitivity to CTP, Etop and 5-FU (Figure 6b), which was associated with decreased cell viability evident by increased PI+/Annexin V- populations, HMGB1 release, and ATP depletion (Figures 6c–e). Treatment of Nec-1, but not z-VAD, reduced 5-FU-induced PI+/Annexin V- populations (Figure 6f). Similar findings were observed in HCT116 C3KD cells (Supplementary Figures S4A and C).

RIP3 is a well-established target of RIP1 to mediate necrosis upon activation of TNF receptor or TLR3.^14, 15, 23^ However, HCT116, C3KO, or RKO cells had little, if any, expression of RIP3 protein or transcript (Supplementary Figures S5A and S5B). We confirmed that RIP3-expressing HT29 cells,^14, 15^ but not C3KO cells, undergo necrosis with TNF-*α*, Smac mimetic and z-VAD treatment (TSZ) (Supplementary Figure S5C). Forced RIP3 expression alone increased cell death in C3KO cells, which did not further increase with TSZ treatment (Supplementary Figure S5D). Furthermore, 5-FU-induced HMGB1 release or cell loss in C3KO cells was not blocked by the RIP3 inhibitor GSK'872^23^ (Supplementary Figures S5E and S5F), or associated with RIP3-mediated MLKL phosphorylation (serine 358)^15^ (Supplementary Figure S5G). 5-FU-induced HMGB1 release was blocked by TNF-*α* antibody in C3KO cells (Supplementary Figure S5H), supporting that TNF-*α* promotes cell killing after severe DNA damage.^24^ Interestingly, TCGA data indicated little or no *RIP3* expression in a substantial fraction of colon cancers compared with normal tissues (*N*=304) (Supplementary Figure S6). These findings strongly argue that targeting caspase-3 might be therapeutically explored in cancer cells to induce RIP3-independent necrosis without blocking apoptosis.

### The role of ROS in 5-FU-induced necrosis

**Figure 7:**
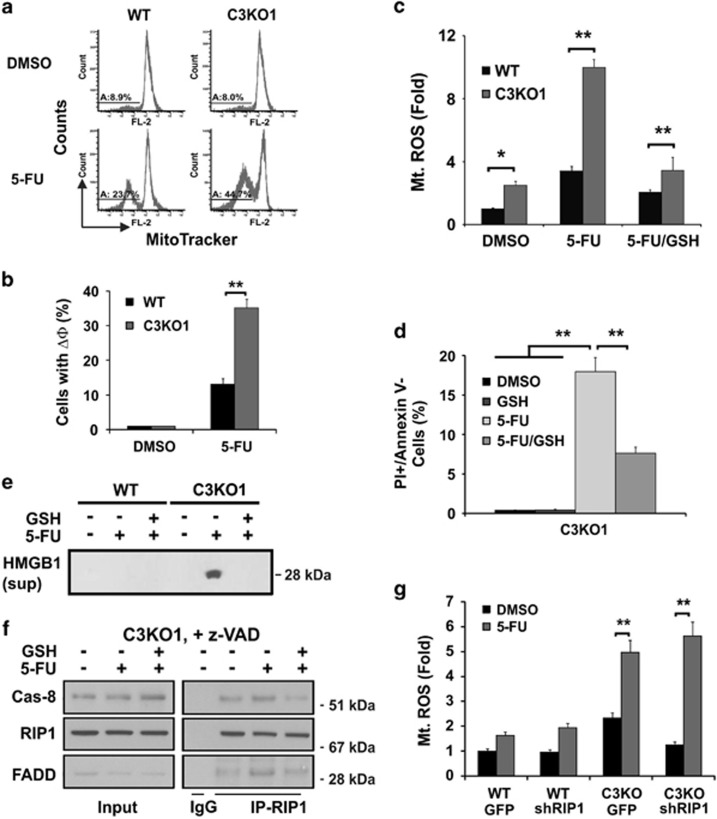
5-FU-induced necrosis in C3KO cells is ROS-dependent. HCT116 WT and C3KO cells were treated with DMSO or 5-FU (50 *μ*g/ml). (**a**, **b**) Mitochondrial outer membrane potential measured by MitoTracker Red CMXRos at 48 h. (**c**) Production of mitochondrial reactive oxygen species (Mt. ROS) measured by mitoSox at 24 h with or without glutathione (GSH, 10 *μ*M). (**d**) Fractions of PI+/Annexin- cells treated as in (**c**). (**e**) Levels of HMGB1 in the medium from cells treated as in (**c**). (**f**) The effects of GSH on the caspase-8/RIP1/FADD complex at 24 h in the presence of z-VAD (20 *μ*M). (**g**) 5-FU induced production of mitochondrial reactive oxygen species (Mt. ROS) at 24 h in control (GPF) or *RIP1* knockdown (shRIP1) WT and C3KO cells. (**b**–**d** and **g**) Data are the mean+S.E.M. of triplicate wells. *P <0.05, **P<0.01

Reactive oxygen species (ROS) has been implicated in necrosis, and mitochondria have a key role in ROS production.^14^ Compared with the controls, C3KO or C3KD HCT116 and RKO cells showed exacerbated mitochondrial dysfunction after 5-FU treatment as indicated by decreased mitochondrial outer membrane potential and production of mitochondrial ROS, and only slightly elevated basal ROS levels (Figure 7c; Supplementary Figures S7A and S7B). ROS scavenger glutathione significantly reduced 5-FU-induced mitochondrial ROS production, HMGB1 release, PI+/Annexin V- populations (Figures 7c and e), and importantly assembly of the z-VAD-resistant caspase-8/RIP1/FADD complex in C3KO cells (Figure 7f). *RIP1* KD did not affect 5-FU-induced ROS production (Figure 7g), or activation of caspase-7 (Supplementary Figure S3). These data support a model in which that ROS, perhaps resulting from mitochondrial damage, acts upstream of RIP1 in DNA damage-induced necrosis (Supplementary Figure S7C).

## Discussion

Oncogenic alterations often render cancer cells resistant to apoptosis,^2, 3, 25^ activation of alternative cell death pathways might therefore provide new therapeutic options. Our work demonstrates that caspase-3 loss sensitizes colon cancer cells to genotoxic agents by promoting RIP1-, pro-caspase-8-, and ROS-dependent necrosis, without blocking apoptosis (Supplementary Figure S7C). This form of necrosis does not require RIP3 or co-administration of z-VAD. Our studies show that pro-casapse-8 promotes necrosis following DNA damage in cancer cells, a role different from suppressing RIP3-dependent necrosis in development. RIP3 expression is reduced or absent in a significant fraction of colon cancers (Supplementary Figure S6), and lung cancer cells,^26^ whereas activated caspase-3 levels, not caspase-3 expression,^19, 27^ were reported to be associated with poor prognosis in certain types of cancer.^28^ Therefore, targeting caspase-3 might be a novel approach to enhance the killing of RIP3-deficient cancer cells by DNA-damaging agents.

It is not surprising that caspase-3 or RIP1 is dispensable in DNA damage-induced apoptosis owing to activation of other caspases or p53-independent NF-κB/TNF-*α* feed forward loop (this study).^24^ However, it is surprising that RIP3 is dispensable in DNA damage-induced necrosis given its well-established role in that induced by TNF-*α* or TLR3 activation.^7, 14, 15, 23^ Our data suggest an interesting possibility that mitochondrial damage and ROS production^29^ control the assembly of the caspase-8/RIP1/FADD complex, therefore both apoptotic and necrotic cell death (Supplementary Figure 7f). Potential roles of redox-sensitive modification of death receptor 5^30^ or RIP1, and downstream mediators such as MLKL and PGAM5 in RIP3-independent necrosis clearly warrant further studies (Supplementary Figure S7C).

Non-overlapping functions of RIP1 and RIP3, or caspase-3 and caspase-8 are well-supported by genetic studies. *RIP1* KO, but not *RIP3* KO, mice have profound immune and survival defects.^31, 32^ Caspase-3-deficient worms or mice had limited apoptotic deficiencies, but were sensitized to certain stress associated with inflammation.^6, 33, 34^ In contrast, genetic ablation of *Casapse-8* leads to RIP3-dependent necrosis and embryonic lethality.^10, 11^ Our data show that caspase-3 regulates the binding of RIP1 to pro-caspase-8 to suppress necrosis in cancer cells lacking RIP3 expression, but is dispensable for apoptosis, suggesting critical differences in cell death regulation in normal *versus* cancer cells, and a potential role of caspase-3 in linking cell killing and innate immunity, that is, via HMGB1 release.^6^ Further investigation will be required to better understand these interactions and their significance in therapeutic responses, while controlled necrosis might be therapeutically beneficial if it does not provoke frank and dangerous systemic inflammation. Cancer cells and mice with knock-in of catalytic or processing deficient caspase-3 or -8 alleles could be very useful.

The z-VAD-resistant pro-caspase-8/RIP1/FADD complex formed after DNA damage described in this study supports that the ripoptosome, a caspase-8 and RIP-containing complex, is capable of promoting apoptosis and necrosis.^21, 35^ Therefore, impaired caspase-8 activation in C3KO cells likely contributes to necrosis by maintaining pro-caspase-8 and RIP1 levels. Further, z-VAD clearly inhibits binding or non-catalytic activities of caspases (Figure 4c). This is reminiscence of necrostatins that can inhibit kinase-dependent and -independent RIP1 functions via conformational changes.^36, 37^ These findings caution careful interpretation of studies using small molecule inhibitors and the importance of genetic models.

In summary, we have shown that genetic ablation of caspase-3 in colon cancer cells increases sensitivity to DNA-damaging agents through RIP1-dependent necrosis without compromising apoptosis. Therefore, pharmacological manipulation of caspase-3 may provide a novel approach to enhance the killing of chemoresistant cancer cells.

## Materials and Methods

### Cell culture and drug treatment

Human colorectal cancer cell lines, including HCT116, HT29, and RKO and embryonic kidney 293T cells were obtained from the American Type Culture Collection (Manassas, VA, USA). RKO, HT29, HCT116, and derivative cell lines were cultured in McCoy's 5A modified media (Life Technologies, Grand Island, NY, USA) at 37 °C/5% CO_2_. 293T cells were maintained in DMEM (Life Technologies) media at 37 °C/5% CO_2._ All media were supplemented with 10% FBS and 1% penicillin–streptomycin solution (Sigma, St. Louis, MO, USA). Unless otherwise noted all drugs were reconstituted in dimethyl sulfoxide (DMSO) and used as follows: 50 *μ*g/ml 5-FU, 100 *μ*M Etop, 750 nM CPT, 15 *μ*M Nec-1 (Sigma), 2 *μ*M, NSA, 3 *μ*M GSK'872 (Calbiochem, Billerica, MA, USA), and 20 *μ*M Z-VAD-FMK (z-VAD) (BaChem, Torrance, CA, USA), 200 nM Smac mimtic TL32711 (TetraLogic, Malvern, PA, USA) (with up to 1% DMSO in medium). Human recombinant TNF-*α* (Peprotech, Rocky Hill, NJ, USA) was used at 10 ng/ml and anti-human TNF-*α* antibody (Cenocor Ortho Biotech Inc, Malvern, PA, USA) was used at 100 ng/ml.

### Targeting *Caspase-3* in HCT116 cells

Gene targeting vectors were constructed by using the rAAV system as previously described.^38^ In brief, two homologous arms (1.334 and 1.411 kb, respectively) flanking exons 5 and 7 of *caspase-3*, along with a neomycin-resistant gene cassette (*Neo*), were inserted between two Not I sites in the AAV shuttle vector pAAV-MCS (Stratagene, La Jolla, CA, USA). Packaging of rAAV was performed by using the AAV Helper-Free System (Stratagene) according to the manufacturer's instructions. HCT116 cells containing two copies of WT *Casapse-3* were infected with the rAAV and selected by G418 (0.5 mg/ml, Mediatech, Manassas, VA, USA) for 3 weeks. Drug-resistant clones were pooled and screened by PCR for targeting events. Prior to targeting the second allele, the *Neo* cassette, which is flanked by Lox P sites, was excised from several heterozygous clones by infection with an adenovirus expressing Cre recombinase (Ad-Cre).^38^ Single clones were screened by PCR for *Neo* excision, and positive clones from independent heterozygotes were infected again with the same *caspase-3*-targeting construct. After the second round, *Neo* was again excised by Ad-Cre infection, and gene targeting in *caspase-3* KO cells was verified by PCR and western blotting. Primer sequences used are listed in Supplementary Table S1.

### *Caspase-3* and *RIP1* KD

Stable KD cells were generated using lentivirus expressing gene-specific shRNA (Supplementary Table S1). KD was determined by quantitative reverse transcriptase polymerase chain reaction (RT-PCR) and confirmed by western blotting. Stable GPF-expressing cells were used as the control for all experiments. Details on the collection of lentiviral particles, lentiviral transduction, and isolation of stable KD clones are found in supplemental materials.^39^

*Caspase-3* KD: lentiviral particles were generated by co-transfection of four plasmids into 293T cells using FuGene 6 Transfection reagent, including Control plasmid (pLK01.GFP-puro) or pLK01.CASP3-sh1 together with pMD2.g (VSVG), pVSV-REV and pMDLg/pRRE. *RIP1* KD: lentiviral particles were generated by co-transfection of four plasmids Lipofectamine 2000 (Invitrogen, Grand Island, NY, USA) into 293T cells, including pLK shRIP1 (Open Biosystems, Lafayette, CO, USA), pMD2.G, pMDLg-pRRE, and pRSV-Rev.

### Other methods

Detailed methods and references are found in supplemental materials on RT-PCR, immunoblotting, IP,^40^ transfection, and RIP1 and RIP3 expression constructs, cell viability, mitochondrial ROS, transmission EM, xenografts,^41, 42^ and histological and staining.^43^

### Statistical analysis

Statistical analyses were carried out using GraphPad Prism IV software. *P-*values were calculated by the student's *t*-test and were considered significant if *P* <0.05. The means+S.E.M. of a representative experiment with triplicates are displayed in the figures. The experiments, except for the xenografts studies, were repeated at three different occasions with similar results.

## Abbreviations

5-FU: 5-fluorouracil
AAV: recombinant adeno-associated virus (rAAV)
CPT: camptothecin
DR5: death receptor 5
Etop: etoposide
FADD: Fas (TNFRSF6)-associated via death domain
HMGB1: high mobility group box 1
MLKL: mixed lineage kinase domain-like protein
Nec-1: necrostatin-1
NSA: necrosulfonamide
PGAM5: phosphoglycerate mutase family member 5
RIP1: receptor (TNFRSF)-interacting serine–threonine kinase 1
RIP3: receptor (TNFRSF)-interacting serine–threonine kinase 3
ROS: reactive oxygen species
shRNA: short-hairpin RNA
SMAC: second mitochondria-derived activator of caspase
TNF-*α*: tumor necrosis factor, alpha
z-VAD: Z-VAD-FMK
