# Supplementary material for: Loss of Caspase-3 sensitizes colon cancer cells to genotoxic stress via RIP1-dependent necrosis
Source: Cell Death Dis. 2015 Apr 23;6(4):e1729–. doi: 10.1038/cddis.2015.104 (PMC4650537; doi:10.1038/cddis.2015.104)

Supplemental material and methods

Caspase-3 and *RIP1* Knockdown

Stable knockdown cells were generated using lentivirus expressing gene specific shRNA (Table S1). Knockdown was determined by quantitative RT-PCR and confirmed by western blotting. Stable GPF expressing cells were used as the control for all experiments. Details on the collection of lentiviral particles, lentiviral transduction and isolation of stable knockdown clones are found in supplemental materials [1]

Caspase-3 knockdown. Lentiviral particles were generated by co-transfection of 4 plasmids into 293-FT cells using FuGene 6 Transfection reagent, including Control plasmid (pLK01.GFP-puro) or pLK01.CASP3-sh1 together with pMD2.g (VSVG), pVSV-REV and pMDLg/pRRE]. The collection and isolation of lentiviral particles, and lentiviral transduction and screening of most effective CASP3 shRNA was carried out as described previously [1]. The CASP3-specific shRNA (shRNA2, table S1) was used in HCT116, RKO and FADD KO cells. In brief, cells were plated and allowed to grow until 40% confluent. Cell media was replaced with a 1:1 dilution of lentivirus containing CASP3-shRNA2 and McCoy's 5A media supplemented with 8µg/ml polybrene (Millipore). Cells were then incubated at 32°C/5% CO_2 for 16 hours. Cell media was removed and replaced with fresh McCoy's 5A and cells were incubated for 8 hours at 37°C/5% CO_2. This process was repeated a second time before putting the cells permanently at 37°C/5% CO_2 under puromycin (2 µg/ml) selection, and single clones were isolated by limiting dilution. Knockdown of *CASP3* was determined by quantitative RT-PCR using an Applied Biosystems StepOnePlus system using the Applied Biosystems Taqman® Gene Expression Cells-to-CT kit [1], and validated by western blotting.

RIP1 knockdown. Lentiviral particles were generated by co-transfection of 4 plasmids Lipofectamine 2000 (Invitrogen) into 293-FT cells, include pLK shRIP1 (Open Biosystems), pMD2.G, pMDLg-pRRE and pRSV-Rev. In brief, 293T cells were plated in 12 well format and allowed to grow to 90% confluence, and transfected with 4 plasmids with Lipofectamine 2000 (Invitrogen), including pLK shRIP1 (Open Biosystems), pMD2.G (0.5 μg), pMDLg-pRRE (0.5 μg) and pRSV-Rev (0.5 μg) according to the manufacturer's instructions. Sequence data is provided in Table S1. 48 hours after transfection, the viral supernatant was harvested with a syringe and needle. Supernatant was then passed through a 0.45μm filter and mixed 4:1 with McCoy's 5A containing 8 μg/ml polybrene. Virus/media mixture was then added to HCT116 caspase-3 KO cells for 48 hours. Cells were permanently maintained at 37°C/5% CO_2 under puromycin (2 μg/ml) selection, and single clones were isolated by limiting dilution.

Reverse transcriptase polymerase chain reaction (RT-PCR)

Total RNA was isolated from cells using the RNAgents Total RNA Isolation System (Promega, Madison, Wisconsin, USA) according to the manufacturer's instructions. First strand cDNA was synthesized using Superscript Reverse Transcriptase (Invitrogen) according to the manufacturer's instructions. PCR was performed on a Thermo Scientific Hybaid MultiBlock MBS 0.2S Thermal Cycler using gene specific primers. Primers used are listed in Table S1. PCR products were analyzed by agarose gel electrophoresis.

Immunoblotting

Cells were harvested in RIPA buffer (50 mM Tris pH 7.4, 150 mM NaCl, 1% Triton-X-100, 0.1% SDS, 1% Deoxycholate, 1 mM EDTA) supplemented with protease inhibitor cocktail (Roche

Applied Sciences, San Francisco, CA). Cells were rotated for one hour at 4ºC and then centrifuged at 13,000 RPM for 30 minutes. Protein concentration was determined by the Bradford assay and 40 μg per well was loaded in 10% bis-tris gels (Life Technologies). Gels ran for 40 minutes at 180v in MOPS buffer (Life Technologies). Protein was then transferred to PVDF membranes using a TransBlot SD semi-dry transfer cell (Biorad, Hercules, CA). Membranes were blocked for nonspecific binding with 5% nonfat milk in TBS-T for one hour at room temperature and then incubated in primary antibody overnight at 4ºC. Primary antibodies were used at a 1:1000 dilution and include HMGB1 (ab18256), pMLKL (Ser358) (ab187091) (Abcam, Cambridge, England); FADD (F36620), RIP1 (610458) (BD Biosciences San Jose, CA), Caspase-7 (9492), Caspase-8 (9746), Caspase-9 (9502), p-p53 (9284) (Cell Signaling, Beverly, MA), RIP3 (IMG-5846A) (Imgenex, San Diego, CA); γ-H2AX (07-164) (Millipore); α-Tubulin (CP06), Noxa (OP180), p21 (OP-64MG) (Oncogene Science, Pittsfield, MA); PUMA (3795) [2], cIAP1 (AF8181) (R&D, Minneapolis, MN); HA (sc-805) (Santa Cruz, Santa Cruz, CA); β-Actin (A5441) (Sigma) and Caspase-3 (AAP103E) (Stressgen, San Diego, CA). Following primary antibody hybridization, membranes were washed with TBS-T and incubated in appropriate HRP-conjugated secondary for 1 hour at room temperature. Secondary antibodies used include goat-anti-rabbit (31462), goat-anti-mouse (31432) and mouse-anti-goat (31400) (Pierce, Rockford, IL). Presence of antibody binding was detected using Western Lighting - Plus ECL (Perkin Elmer, Waltham, MA) according to manufactures specifications. Membranes were then exposed on blue X-ray film (Phenix Research Products, Candler, NC) [3].

Constructs and transfection

Full-length human caspase-3 (Addgene # 11813) and catalytically dead human C163A caspase-3 (Addgene# 11814) were obtained from Addgene (Cambridge, MA). Flagged tagged-RIP1 lacking the entire kinase domain rendering it dominant negative [4] was a generous gift from Dr. Junying Yuan at Harvard University. All plasmids were transfected with Lipofectamine 2000 (Invitrogen) according to the manufacturer's instructions. Stable clones were maintained in McCoy's 5A supplemented with 0.4 mg/ml G418 (Gibco, Grand Island, NY). The HA-tagged full length RIP3 expression vector was constructed by cloning RT-PCR products into the pcDNA3.1-N-HA vector (Invitrogen). Sequences used are listed in table S1.

Small-interfering RNA (siRNA) duplexes were obtained from Qiagen (Venlo, Netherlands) and include: caspase-8 and control scrambled siRNA (Table S1). siRNA duplexes were transfected with Lipofectamine 2000 according to the manufacturer's instructions with minor modifications. Briefly, 400 pmols of siRNA duplexes were transfected into cells in 12-well plates for 4 hours, followed by incubation in medium containing 5% FBS for 20 hours, and then drug treatment in complete media containing 10% FBS.

Cell viability

Cells were plated at 20-30% confluence 24 hours prior to treatment. Unless noted otherwise, cells were treated for 48 hours. Cell proliferation was measured using Cell-Titer 96 Aqueous One Solution Cell Proliferation Assay (MTS assay), and ATP levels were measured using Cell-Titer-GLO Luminescent Viability Assay (Promega, Madison, WI) according to manufacturer's recommendations. In addition, attached and live cells were washed 3 times with HBSS after

various treatments, stained with crystal violet dye (Sigma) for 10 minutes, followed by 3 more rinses with HBSS. All experiments were repeated for at least 3 times.

Mitochondrial outer membrane potential

Cells were plated at 20-30% confluence 24 hours prior to treatment. Following treatment, cells were assayed for mitochondrial outer membrane potential by MitoTracker Red CMXRos according to manufacturer's recommendations as previously described [5]. All experiments were repeated for at least 3 times.

Propidium iodide and Annexin V staining

Cells were plated at 20-30% confluence 24 hours prior to treatment. Following 48 hours treatment, cells were trypsinized and spun down at 3,000 RPM (400 g). Cells were then washed in cold PBS and reconstituted in 100µl of Annexin-binding buffer (10 mM HEPES, 140 mM NaCl, 2.5 mM $CaCl_2$, adjusted to pH=7.4) containing 2 µl of Annexin V-Alexa Fluor 488 and 2 µl 50 µg/ml propidium iodide (Invitrogen). Cells were then allowed to incubate in the dark at room temperature for 15 minutes before being diluted with 300 µl Annexin-binding buffer. FL1 and FL2 emission was measured on a C6 flow cytometer (Accuri Cytometers, San Jose, CA). At minimum, 30,000 events per sample were measured, all samples were done in triplicate and all experiments were performed at minimum three times.

Detection of reactive oxygen species

Cells were plated at 20-30% confluence 24 hours before treatment and then treated for 24 hours. Following treatment, cell media was removed and replaced with fresh media containing 2 µM

mitoSox reagents (Life Technologies). Cells were then incubated for 20 minutes at 37ºC/5% CO_2. Following incubation cells were harvested washed once in cold phosphate-buffered saline (PBS) and then reconstituted in cold 1% BSA PBS. Following excitation at 518 nm, 580 nm emission was measured on a C6 flow cytometer (Accuri Cytometers). At minimum, 30,000 events per sample were measured, all samples were done in triplicate and all experiments were performed at minimum three times.

Transmission electron microscopy

Cells grown on tissue culture plasticware were fixed in 2.5% gluaraldehyde in 100 mM PBS (8 gm/l NaCl, 0.2 gm/l KCl, 1.15 gm/l $Na_2HPO_4 \cdot 7H_2O$, 0.2 gm/l KH_2PO_4, pH 7.4) overnight at 4°C. Monolayers were then washed in PBS three times then post-fixed in aqueous 1% osmium tetroxide, 1% Fe_6CN_3 for 1 hr. Cells were washed 3 times in PBS then dehydrated through a 30-100% ethanol series then several changes of Polybed 812 embedding resin (Polysciences, Warrington, PA). Cultures were embedded in by inverting Polybed 812-filled BEEM capsules on top of the cells. Blocks were cured overnight at 37°C, and then cured for two days at 65°C. Monolayers were pulled off the coverslips and re-embedded for cross section. Ultrathin cross sections (60 nm) of the cells were obtained on a Riechart Ultracut E microtome, post-stained in 4% uranyl acetate for 10 min and 1% lead citrate for 7 min. Sections were viewed on a JEOL JEM 1011 transmission electron microscope (JEOL, Peobody MA) at 80 KV. Images were taken using a side-mount AMT 2k digital camera (Advanced Microscopy Techniques, Danvers, MA) [6].

Immunoprecipitation

The protocol was modified from [7]. In brief, following harvesting of cells, 5 mg of protein reconstituted in 1 ml of RIPA in each experiment and used with Invitrogen Protein A and G Dynabead® immunoprecipitation system according to manufacturer's instructions. Antibodies used include Caspase-3 (sc-7148), Caspase-8 (sc-6136) (Santa Cruz) and RIP1 (610458) (BD Biosciences). Resulting precipitates were processed in the same manner as other immunoblots as described above [7].

Xenograft studies

All animal experiments were approved by the University of Pittsburgh Institutional Animal Care and Use Committee. Female 5–6 week-old Nu/Nu mice (Charles River, Wilmington, MA) were housed in a sterile environment with micro isolator cages and allowed access to water and chow *ad libitum*. Mice were injected subcutaneously in both flanks with 4×10^6 WT or *caspase-3*-KO HCT116 cells. After implantation, tumors were allowed to grow 7 days and reach approximately $50mm^3$ before treatment was initiated. Mice were randomized into two groups (n = 9 per group) receiving either vehicle (PBS) or 5-FU (50 mg/kg/d) every other day for 14 days. Detailed methods on tumor measurements, harvests and histological analysis are as described [8-9].

Tissue preparation and analysis of histology and Immunohistochemistry (IHC)

Following sacrifice of mice, tumors were dissected and fixed in 10% formalin for histological analysis as described previously [8-9]. Five-micron (5 μm) sections were used for staining. Histological analysis was performed by hematoxylin and eosin (H&E) staining. Cell death was analyzed by TUNEL staining with the ApopTag Fluorescein In Situ Apoptosis Detection Kit (Chemicon International, Temecula, CA) according to the manufacturer's instructions.

Activated caspase-3 IHC was performed as described [10]. In brief, tissue sections (5µm) were deparafinized, rehydrated through graded ethanol and treated with 3% hydrogen peroxide. Antigen retrieval was performed by boiling the sections for 10 minutes in 0.1 M citrate buffer (pH 6.0) with 1 mM EDTA. Non-specific antibody binding was blocked using 20% goat serum at room temperature for 30 minutes. Sections were incubated overnight at 4◦C in a humidified chamber with 1:100 diluted rabbit-anti-caspase-3 (cleaved, Asp 175) (9661; Cell Signaling Technologies). Sections were then incubated for 1 h at room temperature with biotinylated goat-anti-rabbit secondary antibodies (#31822; Pierce) and developed with an ABC kit and DAB (Vector Laboratories, Burlingame, CA).

Supplemental Tables

Table S1: Primer and shRNA sequences used in experiments

Target	Application	Sequence
Caspase-3	Right Arm Forward 1	5'-CTGGGAAGATAGCAGGGTTTGTGT-3'
	Right Arm Reverse 1	5'-TTTGTGAGCATGGAAACAATACATGT-3'
	Left Arm Forward 1	5'-AGAACTGGACTGTGGCATTGAG-3'
	Left Arm Reverse 1	5'-GCTTGTCGGCATACTGTTTCAG-3'
Neomycin	Forward 1	5'-TCTTGACGAGTTCTTCTGAG-3'
	Reverse 1	5'-TTGTGCCCAGTCATAGCCG-3'
GAPDH	Forward 1	5'-TGCACCACCAACTGCTTAGC-3'
	Reverse 1	5'-GGCATGGACTGTGGTCATGAG-3'
RIP1	Forward 1, RT-PCR	5'-GGCGTCATCATAGAGGAAGG-3'
	Reverse 1, RT-PCR	5'-TGTGTATCACGCCTTTTCCA-3'
RIP2	Forward 1, RT-PCR	5'-GGAATTATCTCTGAACATACCTG-3'
	Reverse 1, RT-PCR	5'-CCTGGACAGAAGGGCATCTAGC-3'
RIP3	Forward 1, RT-PCR	5'-TGCTGGAGGAGAAGTTGAGTTGC-3'
	Reverse , RT-PCR	5'-CTGTTGCACACTGCTTCGTACAC-3'
Control	siRNA	5'-AACGUACGCGGAAUACUUCGA-3'
Caspase-8	siRNA	5'-AAGAGTCTGTGCCCAAATCAA-3'
Control	shRNA	5'-CCGCAGGTATGCACGCGT-3'
RIP1	shRNA	5'-TAAGCTGAAAGAACATGACCT-3'
Caspase-3	**shRNA2 (used)**	**5'-CCTGAGATGGGTTTATGTATA-3'**
RIP3	Expression plasmid F	5'-TTCAAGCTTGATGTCGTGCGTCAAGTTATGGCC-3'
RIP3	Expression plasmid R	5'-CGTTGAGCTGAGTTGCCAGCTGGTCTAAAAGAGTATC-3'

Table S2: Quantification of Caspase-8 and Casapse-3 interacting proteins. The band intensity in IP-Cas-8 and IP-Cas-3 experiments in Figure 4, was quantified by image J. The levels of the bait protein (underlined) were set at 1 to allow calculation of relative abundance f interacting proteins before and after 5-FU treatment in WT or C3KO cells.

Table S2. Quantification of interacting proteins				
**Figure 5A**				
	**WT**		**C3KO1**	
**5-FU**	-	+	-	+
**Caspase-8** (underlined)	1.00	1.00	1.00	1.00
**Caspase-3**	NB	0.10	NB	NB
**RIP1**	0.17	0.21	0.04	0.42
**FADD**	0.38	0.39	0.30	0.54
**Figure 5A**				
	**WT**		**C3KO1**	
**5-FU**	-	+	-	+
**Caspase-8**	0.94	2.11	NB	NB
**Caspase-3** (underlined)	1.00	1.00	NB	NB
**RIP1**	1.06	1.36	NB	NB
**FADD**	0.20	0.33	NB	NB
**Figure 5B**				
	**WT**		**C3KO1**	
**5-FU**	-	+	-	+
**zVAD**	+	+	+	+
**Caspase-8** (underlined)	1.00	1.00	1.00	1.00
**Caspase-3**	NB	NB	NB	NB
**RIP1**	0.26	1.20	0.20	1.46
**FADD**	0.08	0.24	0.06	0.45
**Figure 5B**				
	**WT**		**C3KO1**	
**5-FU**	-	+	-	+
**zVAD**	+	+	+	+
**Caspase-8**	NB	NB	NB	NB
**Caspase-3** (underlined)	1.00	1.00	NB	NB
**RIP1**	NB	NB	NB	NB
**FADD**	0.09	0.08	NB	NB
**Target (bait) protein is underlined.**				
**NB = No detectable binding of proteins**				

Table S3: Quantification of 5-FU induced caspase-8 interactome with or without z-VAD.

The levels of bait protein (pro-caspase 8) were set at 1 to allow calculation of relative enrichment (fold) of interacting protein RIP1 and FADD in the 5-FU treated WT or C3KO cells over control cells using values in Table S2. The data from cells without (left) or with (right) z-VAD treatment are included.

Table S3.Quantification of 5-FU- induced caspase-8 interactome					
	Without z-VAD			with z-VAD	
	WT	C3KO1		WT	C3KO1
RIP1	1.23	10.47		4.6	7.3
FADD	1.01	1.82		3.0	7.5

Supplemental References

1. Tang J, Goellner EM, Wang XW, Trivedi RN, St. Croix CM, Jelezcova E *et al.* Bioenergetic Metabolites Regulate Base Excision Repair-Dependent Cell Death in Response to DNA Damage. *Molecular Cancer Research* 2010; **8**(1)**:** 67-79.

2. Yu J, Wang Z, Kinzler K, Vogelstein B, Zhang L. PUMA mediates the apoptotic response to p53 in colorectal cancer cells. *Proceedings of the National Academy of Sciences of the United States of America* 2003; **100**(4)**:** 1931-1936.

3. Kohli M, Yu J, Seaman C, Bardelli A, Kinzler KW, Vogelstein B *et al.* SMAC/Diablo-dependent apoptosis induced by nonsteroidal antiinflammatory drugs (NSAIDs) in colon cancer cells. *Proc Natl Acad Sci U S A* 2004; **101**(48)**:** 16897-902.

4. Christofferson D, Li Y, Hitomi J, Zhou W, Upperman C, Zhu H *et al.* A novel role for RIP1 kinase in mediating TNFα production. *Cell death & disease* 2012; **3**.

5. Sun Q, Sakaida T, Yue W, Gollin SM, Yu J. Chemosensitization of head and neck cancer cells by PUMA. *Mol Cancer Ther* 2007; **6**(12)**:** 3180-8.

6. Wickline E, Du Y, Stolz D, Kahn M, Monga S. γ-Catenin at adherens junctions: mechanism and biologic implications in hepatocellular cancer after β-catenin knockdown. *Neoplasia (New York, N.Y.)* 2013; **15**(4)**:** 421-434.

7. Ming L, Wang P, Bank A, Yu J, Zhang L. PUMA Dissociates Bax and Bcl-X(L) to induce apoptosis in colon cancer cells. *The Journal of biological chemistry* 2006; **281**(23)**:** 16034-16042.

8. Sun Q, Ming L, Thomas SM, Wang Y, Chen ZG, Ferris RL *et al.* PUMA mediates EGFR tyrosine kinase inhibitor-induced apoptosis in head and neck cancer cells. *Oncogene* 2009; **28**(24)**:** 2348-57.

9. Dudgeon C, Wang P, Sun X, Peng R, Sun Q, Yu J *et al.* PUMA induction by FoxO3a mediates the anticancer activities of the broad-range kinase inhibitor UCN-01. *Molecular cancer therapeutics* 2010; **9**(11)**:** 2893-902.

10. Leibowitz BJ, Qiu W, Liu H, Cheng T, Zhang L, Yu J. Uncoupling p53 functions in radiation-induced intestinal damage via PUMA and p21. *Mol Cancer Res* 2011; **9**(5)**:** 616-25.

Supplemental Figure Legends

Figure S1. Enhanced responses of *caspase-3* KO tumors to 5-FU *in vivo*. (A) Schematic representation of *caspase-3* genomic locus and the targeting strategy. NeoF, NeoR, P1 and P2 are PCR primers (Table S1) used to identify and confirm (P1/P2) knockout clones. **(B)** H&E staining of HCT116 WT tumors after the last treatment as in Figure 2. **(C)** H&E staining of C3KO tumors after the last treatment. Scale bars, 200 µm.

Figure S2. DNA damage response and apoptosis in *caspase-3* KO cells. HCT 116 and C3KO cells were treated with 5-FU (50 µg/ml). **(A)** The indicated DNA damage response proteins were analyzed by western blotting at 24h. **(B)** Flow cytometry profiles of indicated isogenic cell lines stained with PI and Annexin V at 48h. **(C)** Fractions of PI+ cells 48h with or without z-VAD (20 µM). **p<0.01. **(D)** Levels of HMGB1 in the medium of WT or two C3KO clones 48h after CPT (500 nM) or Etoposide (50 µM) treatment. **(E)** Fractions of Annexin PI+/V- (non apoptotic) cells treated as in **D**. Data are the mean + SEM of triplicate wells. **p<0.01.

Figure S3. Inhibition of RIP1 or MLKL does not block 5-FU-induced processing of caspase-7 in C3KO cells. The processing of caspase-7 detected by western blotting from indicated cells 48h after 5-FU (50 µg/ml) treatment, **(A)** with or without RIP1 targeting agents, Nec-1 (15 µM, and **(B)** With or without MLKL inhibitor NSA (2 µM).

Figure S4. 5-FU induces necrosis in *caspase-3* KD HCT 116 cells. (A) Stable *GFP* or *caspase-3* knockdown (shC3) HCT 116 cells confirmed by western blotting. **(B)** Levels of HMGB1 in the

medium from cells 48h after 5-FU (50 μg/ml) treatment. **(C)** Fractions of PI+/Annexin V- cells treated as in **B**. Data are mean + SEM of triplicate wells. **p<0.01.

Figure S5. 5-FU induces RIP3-independent necrosis in CRC cells. **(A)** RIP3 protein levels in the indicated CRC lines analyzed by western blotting. HT29 cells were used as a positive control. **(B)** Levels of *RIP1, 2 and 3* transcripts were analyzed by RT-PCR 48h after DMSO or 5-FU treatment. (**C)** Levels of HMGB1 in the medium 48h after TSZ (TNF-α 10 ng/ml, Smac mimtic TL32711 200 nM, and z-VAD, TSZ, 20 uM). treatment in HT29 and C3KO cells. (**D)** Fractions of PI+ (dying) cells treated as in **C**. Data are the mean + SEM of triplicate wells. **p<0.01. The expression HA-RIP3 in C3KO1 cells was confirmed by western using antibodies against RIP1 or the HA tag. The HT29 cells were used as a positive control for RIP1. **(E)** Adherent cells in two C3KO clones 48h after 5-FU (50 μg/ml) treatment with or without RIP3 inhibitor, GSK'872 (3 μM). (**F)** Levels of HMGB1 in the medium 48h from cells treated as in **E.** (**F)** Level of pMLKL1 (S358) at 24h in 5-FU-treated parental (WT), C3KO cells, or TSZ-treated HT29 cells. (**F)** Levels of HMGB1 in the medium 48h from 5-FU treated cells with or without TNFα antibody (100 ng/ml, added 1h before 5-FU).

Figure S6. Reduced *RIP3* expression in colon cancer. Heatmap display of *RIP3* expression in colon cancer and normal tissues based on TCGA RNAseq data. N=304. White, average expression, Blue, low expression, red, high expression.

Figure S7. 5-FU-induced necrosis in *caspase-3* deficient cells. The control (GFP) or *Casapse-3* knockdown (shC3) cells were treated with 5-FU (50 μg/ml) for 24h. The production of

mitochondrial ROS (Mt. ROS) was measured in HCT 116 **(A)** and RKO cells **(B)**. Data are the mean + SEM of triplicate wells. **p<0.01. (**C**) A model. Loss of caspase 3 stabilizes a necrotic complex downstream of the TNF receptor family by preventing RIP1 and pro-casapse-8 cleavage. Mitochondrial permeability and ROS production might be additionally regulated by p53 or MLKL to promote cell killing.

Figure S1 . Related to Figs 1 and 2

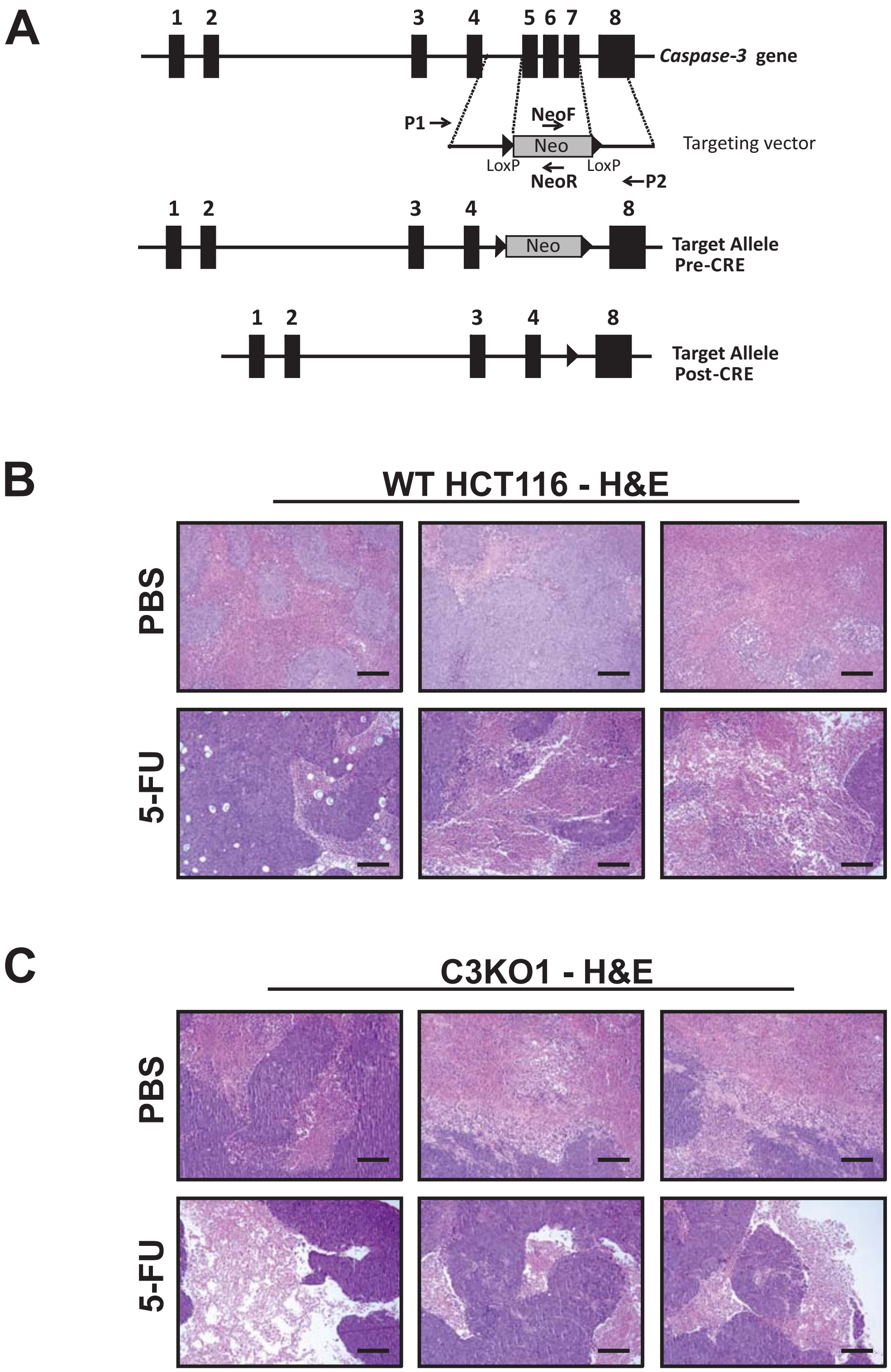

Figure S2. Related to Fig 3.

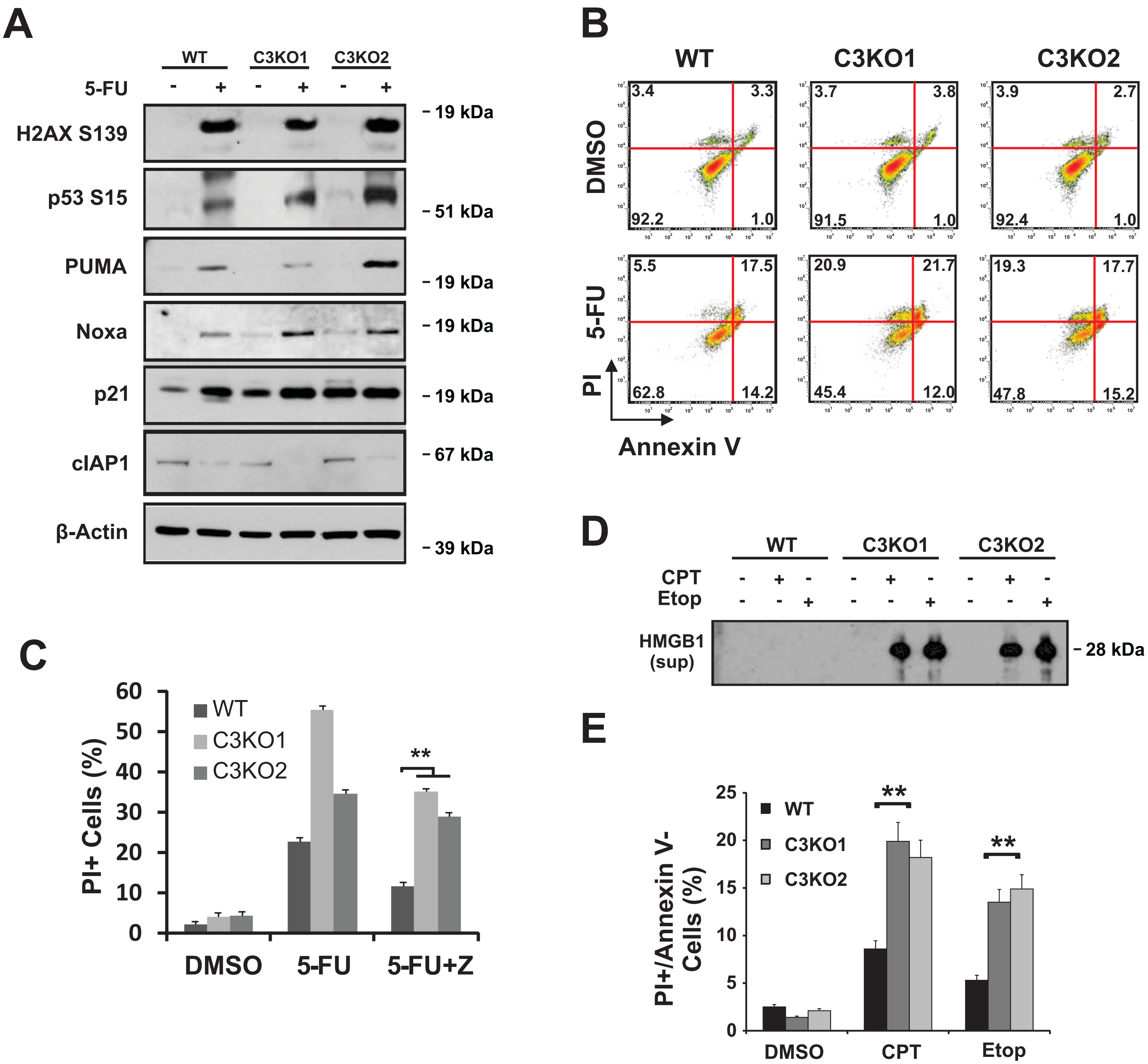

Figure S3. Related to Fig.5

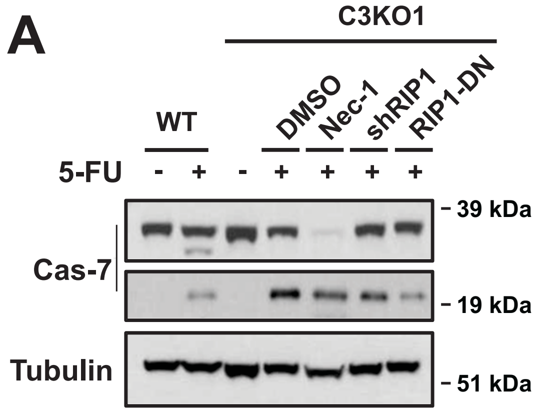

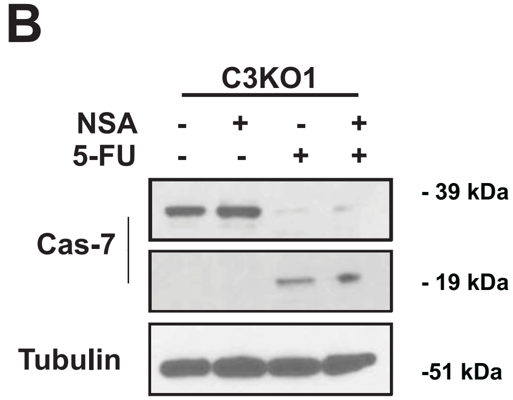

Figure S4, related to Fig.6

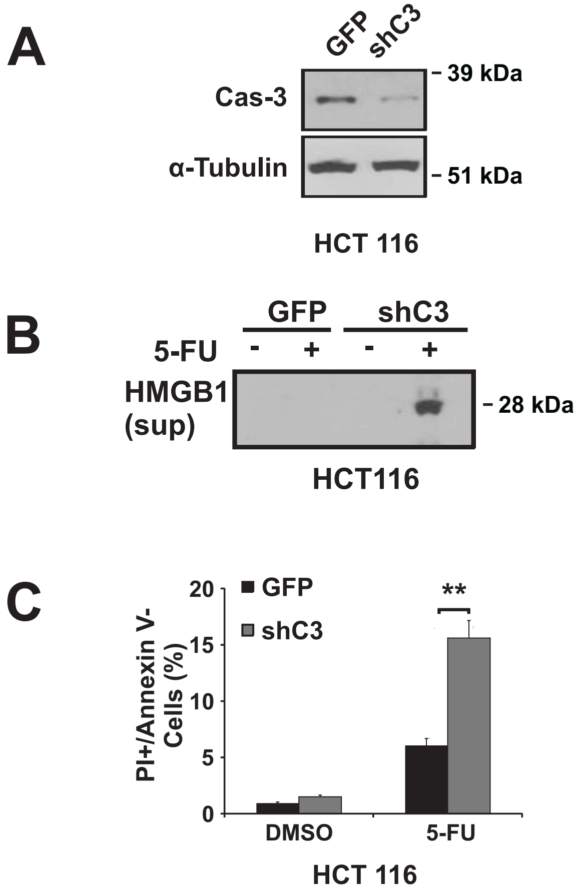

Brown et al. Figure S5, related to Fig. 6

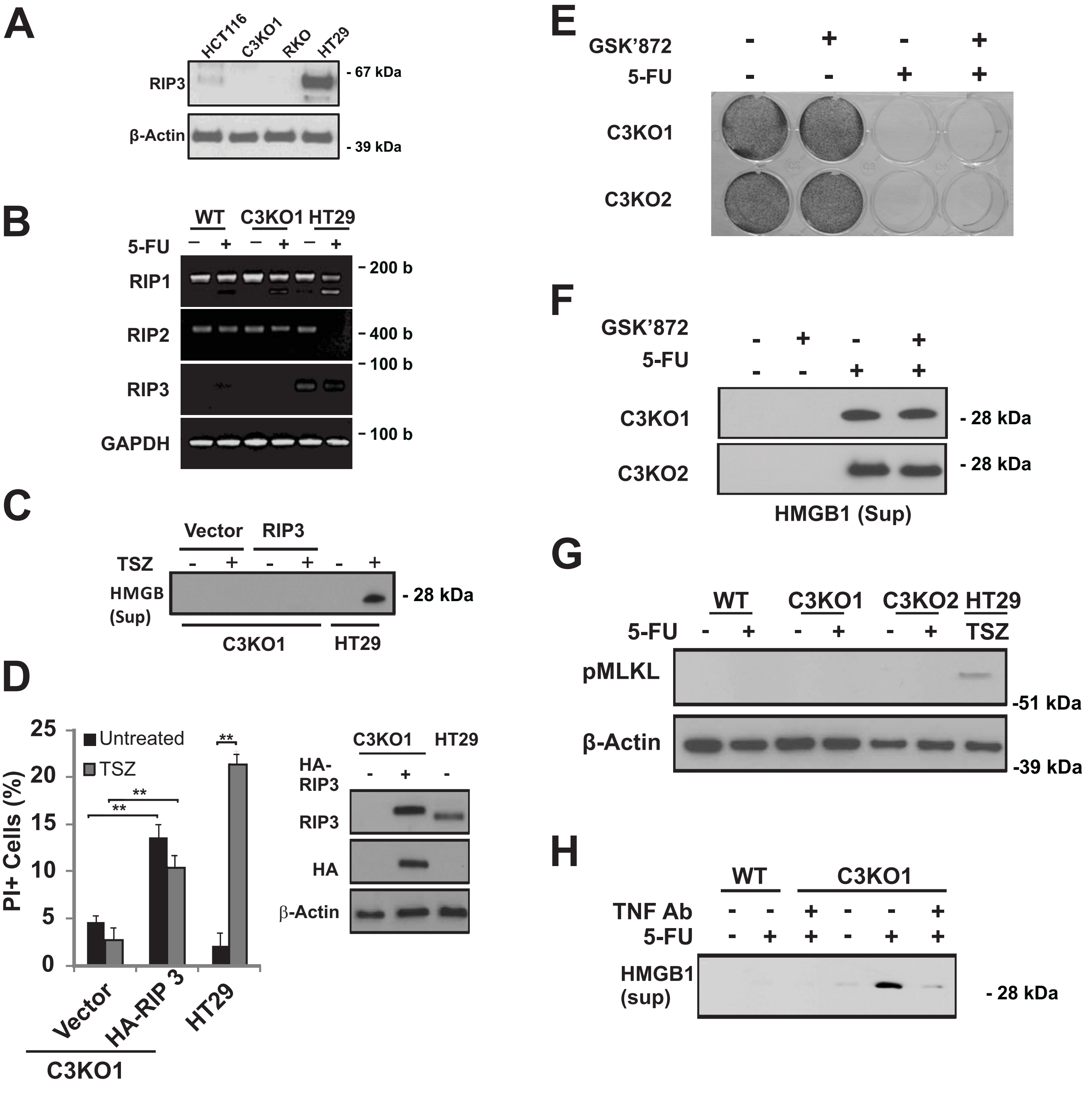

Figure S6, related to Fig. 6

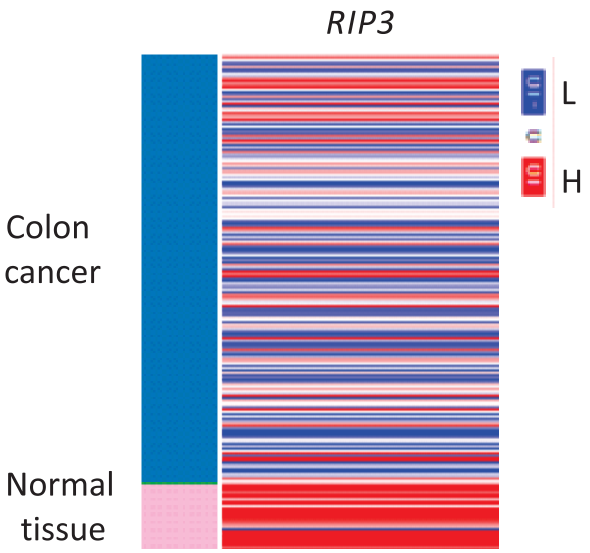

TCGA colon adenocarcinoma (COAD) gene expression By RNAseq , N=304

Figure S7, related to Fig. 7

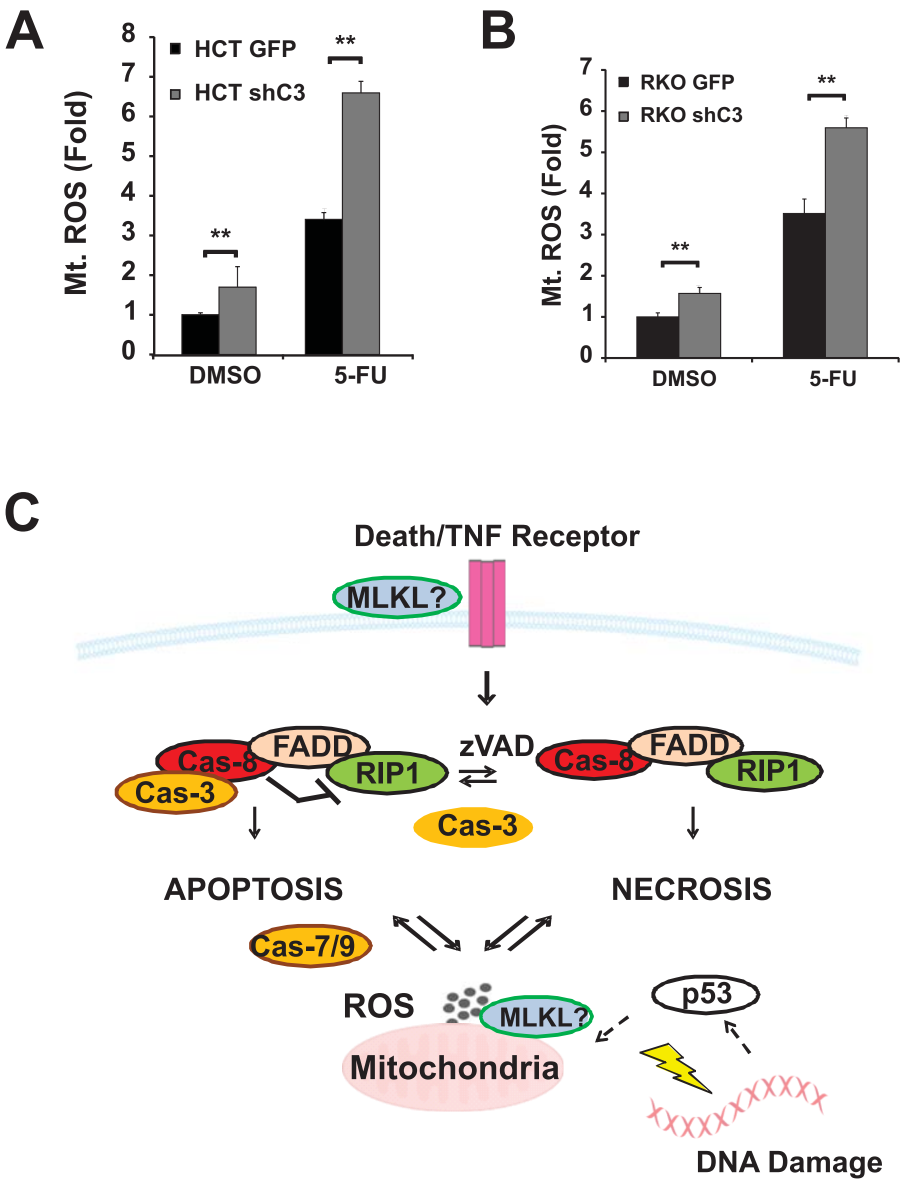

Supplement: Supplementary Information [file cddis2015104x1.pdf]
